# Physicochemical and rheological characterization of pectin‐rich polysaccharides from *Gardenia jasminoides* J. Ellis flower

**DOI:** 10.1002/fsn3.1612

**Published:** 2020-05-06

**Authors:** Qi Chen, Gang Xue, Qinxue Ni, Yan Wang, Qianxin Gao, Youzuo Zhang, Guangzhi Xu

**Affiliations:** ^1^ Zhejiang Provincial Key Laboratory of Agricultural Product Quality Improvement Technology Science School of Agriculture and Food Science Zhejiang Agriculture and Forestry University Zhejiang China

**Keywords:** antioxidant activity, chemical composition, *Gardenia jasminoides* J. Ellis, pectin, rheological properties

## Abstract

Gardenia (*Gardenia jasminoides* J. Ellis) is regarded as an edible medicine plant in China. Here, gardenia flower polysaccharide fraction (GFPF) was extracted by water at 90°C and its chemical composition, rheological properties, and antioxidant activities of GFPF were investigated. The GFPF extraction yield was 18.04 ± 1.81% (W/W) and mainly comprised neutral sugars (46.83 ± 3.14%), uronic acid (35.21 ± 0.17%), protein (1.63 ± 0.34%), and total phenol (9.49 ± 0.08 mgGAE/g). Galacturonic acid (41.05 ± 0.59%) was the main monosaccharide, and galactose, glucose, arabinose, rhamnose, xylose, mannose, and glucuronic acid were also detected in GFPF. Its degree of esterification was 32.76 ± 1.52%. FT‐IR spectra analysis showed a similar absorption pattern between GFPF and pectin from apple. The results suggested that GFPF was low methoxy pectin. Thermogravimetric analysis and zeta potential analysis indicated that the pectin was stable under high temperature and alkaline condition. Steady rheology showed that the GFPF dispersion was a shear thinned pseudoplastic fluid with high apparent viscosities at concentration above 2%. The degree of pseudoplasticity of the solutions increased with the concentrations increased and the temperatures decreased. DPPH and ABTS free radical scavenging assay indicated that GFPF had relatively high antioxidant activity. The results showed that gardenia flower was rich in pectin polysaccharides with low methoxy pectin. It had high apparent viscosities at concentration above 2% and had good antioxidant activity. The data suggested that GFPF can be a new resource of low methoxy pectin with potential application as thicker or gelling agents in food industry.

## Graphical abstract

Gardenia jasminoides flower polysaccharide fraction was extracted by hot water, and the yield was 18.04 ± 1.81% (W/W). The polysaccharides were rich in low methoxyl pectin and had high antioxidant activity. The rheological properties of Gardenia jasminoides flower polysaccharide fraction were evaluated.

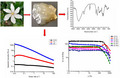

## 1 INTRODUCTION

*Gardenia jasminoides* J. Ellis, a flowering plant belonging to the family of Rubiaceae, is widely distributed in the south of China, which is traditional regarded as an edible medicine plant in China. Its fruit contains many functional components such as crocin, geniposide, genipin, and phenolic compounds (Yin & Liu, 2018) and has traditionally been used as a folk medicine in China and many East Asian countries for thousands of years with many biological activities, such as antioxidant activities, improving insulin sensitivity and antidiabetes, anti‐inflammatory activity, and antidepressant activity (Shan et al., 2017;Xiao, Li, Wang, & Ho, 2017). Besides the application in folk medicine, gardenia fruit is used as a natural food colorant and its flower is also used as edible vegetable in China (Wang et al., 2017). Recently, gardenia flower was also reported to have a wide range of bioactive phytochemicals, such as iridoids and terpenoids, which may provide desirable health benefits with anticancer and antioxidant activities (Zhang et al., 2017, 2018). By contrast, the content and bioactivity of other components of flower, such as nonstarch polysaccharides (NSPs), still did not attract much attention yet.

Pectin, an structurally heterogeneous acid polysaccharides, is one of the major NSPs in plant cell wall (Marić et al., 2018). Due to its strong hydrophilic character and favorable rheological properties, pectin is widely using in food industry as a gelling agent, emulsifier, stabilizer, and food thickener (Chan, Choo, Young, & Loh, 2017). Pectin is often esterificated by methyl groups (at C‐6) and/or acetyl groups (at O‐2 and/or O‐3) at galacturonic acid residues. According to degree of esterification (DE), the pectin is divided into two groups: low methoxy pectin (LMP, DE < 50%) and high methoxy pectin (HMP, DE > 50%) (Dranca & Oroian, 2018). The degree of esterification offers pectin with the distinct gels formation characteristics. HMP forms a gel in an acidic environment (pH < 3.5) or in the presence of a high concentration of low molecular weight cosolutes, such as sucrose (more than 55%) (Giacomazza, Bulone, San Biagio, & Lapasin, 2016). LMP can also form gel in an acidic condition (pH < 3.3). Moreover, it can form gel with a wide pH range when divalent ions such as ionic calcium are present (Han et al., 2017;Yang, Nisar, Liang, et al., 2018). LMP is more preferred in some functional foods.

Commercial pectin is mainly extracted from many cheap resources such as apple pomace (Cho et al., 2019) and citrus peels (Hosseini, Khodaiyan, & Yarmand, 2016), which is mainly HMP. The HMP can be de‐esterificated by chemical or enzymatic methods to produce LMP (Buchholt, Christensen, Fallesen, Ralet, & Thibault, 2004;Wan, Chen, Huang, Liu, & Pan, 2019). However, HMP de‐esterification by using chemical methods may lead to chemical waste‐induced environmental damage and to degrade the pectin resulting in poor thickening or gelling properties (Yoo, Fishman, Savary, & Hotchkiss, 2003). Moreover, HMP de‐esterification by enzymatic methods can increase the product price. Finding new sources of natural LMP has attracted more attention in recent years (Lu et al., 2019;Yuliarti, Chong, & Goh, 2017).

Here, we found that gardenia flower is a new resource for LMP. The aim of this study was to determine the chemical compositions, thermal stability, rheological behaviors, and antioxidant activities of pectin extracted from gardenia. It may provide a foundation for its application in food production.

## 2 MATERIALS AND METHODS

### 2.1 Materials and chemicals

Materials: *G. Jasminoides* J. Ellis flowers were harvested from Zhejiang Province in June 2016. After dried with hot air at 60°C for 24 hr, the flowers were milled using a blender (YB‐1500A), sieved (40 mesh pass), and stored in plastic bags in 4°C before extraction.

Chemicals: Monosaccharide standards（galacturonic acid, glucose, glucuronic acid, mannose, arabinose, rhamnose, xylose, and galactose） were purchased from Tokyo Chemical Industry Development Co., Ltd (Shanghai, China). 2,2'‐Azino‐bis(3‐ethylbenzothiazoline‐6‐sulfonic acid) (ABTS), 3‐phenylphenol (97%), 1‐pheny‐3‐methyl‐5‐pyrazolone (PMP) (99%), and 1,1‐diphenylpicrylhydrazyl (DPPH) were purchased from Sigma‐Aldrich Chemical Co., Ltd. Acetonitrile (HPLC grade) and methyl alcohol (HPLC grade) were purchased from TEDIA Co., Ltd (USA). And all the other chemicals used were of an analytical grade from Aladdin Chemical Reagent Co., Ltd.

### 2.2 GFPF extraction

To remove pigments, lipids, peptides, and other low molecular weight compounds, the milled gardenia flower was washed twice with 85% ethanol overnight at room temperature. The resulting residues were dried at room temperature. To extract GFPF, the dried biomass was mixed with distilled water at a ratio of 1:40(w/v) at 90°C with stirring for 2 hr. The mixture was centrifuged at 5,000 *g* for 20 min. After extraction twice, the supernatants were combined and concentrated by rotary evaporator to 1/2 of original volume at 60°C. The GFPF was precipitated by adding three volumes of ethanol and kept at 4°C overnight. The precipitate was separated by vacuum filtration and dehydrated with ethanol. The resulting GFPF was dried at room temperature in a fume hood. The extract was repeated three times, and the yield was calculated according to the dried biomass obtained after treating the milled sample with 85% ethanol.

(1) $$\text{Yield}\;\left(m/m,\%\right)=\frac{A}{B}\times 100\%$$

where A was the weight of GFPF from gardenia and B was the weight of powder.

### 2.3 Proximate analysis

Neutral sugar content of the GFPF was determined by the phenol–sulfuric acid assay using D‐glucose as a standard (Rover, Johnston, Lamsal, & Brown, 2013). Uronic acid content was determined by a sulfamate/m‐hydroxy‐diphenyl assay using galacturonic acid as a standard (Blumenkrantz & Asboe‐Hansen, 1973). Protein content was determined by Bradford method using BSA as a standard (Bradford, 1976). Total phenol content was determined by the Folin‐phenol assay using gallic acid as a standard (Lapornik, Prošek, & Golc, 2005).

### 2.4 Monosaccharide composition of GFPF

Monosaccharide composition of GFPF was analyzed by reversed‐phase HPLC using PMP as a precolumn derivatization reagent as previously described by (Dai et al., 2010) with some modification. Briefly, the GFPF was hydrolyzed with 4 M TFA at 110°C for 4 hr. The hydrolyzed GFPF or monosaccharide standards (200 μl) were mixed with 0.3 M aqueous NaOH (200 μl) and 0.5 M PMP‐methanol solution of PMP (240 μl). The mixture was maintained at 70°C for 100 min. After cooled to room temperature, it was neutralized with 0.3 mol/L HCl (200 μl) and then extracted with chloroform (1 ml) three times to remove free PMP. The aqueous layer was filtered through a 0.45 μm membrane before HPLC analysis.

Monosaccharide composition analysis was carried out on an Essentia LC‐16 liquid chromatography system by using a reversed‐phase C18 column (Kinetex 5µ XB‐C18 100A 250 × 4.6 mm, Phenomenex). Monosaccharide‐PMP derivatives were eluted isocratically with 83.0% phosphate‐buffered saline (PBS, 0.1  M, pH6.8) and 17.0% acetonitrile (v/v) at a constant flow rate of 1 ml/min with UV detection at 245 nm. The monosaccharide content was calculated according to the calibration curve (peak area concentration) of each monosaccharide standard.

### 2.5 Measurement of the degree of esterification

The DE of GFPF was determined by titration method as described by (Trujillo‐Ramírez et al., 2018). Briefly, 0.1 g GFPF was dissolved into 50 ml distilled water. After adding 3 droplets of phenolphthalein to the solution, it was titrated using sodium hydroxide (0.1 M) until the solution turned pink and the titration volume was recorded as *V*1. And then, 5 ml sodium hydroxide (0.5 M) was mixed in the solution and kept at room temperature for 20 min. Then, 10 ml hydrochloric acid (0.5 M) was added, and the sample was shaken violently until the pink color vanished. The resultant solution was titrated by using 0.1 M sodium hydroxide until the solution turned pink, and the volume was recorded as *V*2. The DE was calculated using the formula:

(2) $$\text{DE}\;\left(\%\right)=\frac{V1}{V1+V2}\times 100\%$$

### 2.6 FT‐IR analysis

The structural characteristics of GFPF were investigated using infrared spectroscopy. The extracted pectin was analyzed by FT‐IR (IR Prestige‐21, Shimadzu, Japan) using wavelength range from 500 cm^−1^ to 4,000 cm^−1^ on KBr disks with a 90:10 KBr/pectin ratio. Pectin from apple (Sigma‐Aldrich, NO. 93854) was used as reference.

### 2.7 Zeta potential measurement

The aqueous dispersion of GFPF (1 mg/ml) was adjusted by 0.1 M NaOH or HCl to pH 2.0–11.0. The zeta potential was analyzed by using high‐resolution zeta potential analyzer (Brookhaven ZetaPALS) at 25°C. Each sample was measured three times, and all measurements were carried out at 25°C.

### 2.8 Molecular weight analysis

Molecular weight of GFPF was estimated by gel permeation chromatography (GPC) system (Elite P230, Dalian, China) using PL aquagel–OH MIXED column (7.5 × 300 mm) and refractive index detector (RI‐201H). GFPF solution was filtered through a 0.22 μm membrane filter and injected to the column through 50 μL loop. The mobile phase was 0.1 M NaNO3 with a flow rate of 1 ml/min. Calibration plot was established using standard dextran samples with known molecular weights. Evaluation of the chromatograms was carried out using size exclusion chromatography software (EC2000 GPC, Elite) to determine the molecular weights of the samples.

### 2.9 Thermogravimetric analysis

The thermal properties of GFPF were evaluated by Thermo Gravimetric Analyzer (*STA 409C, NETZSCH, Germany). The samples (5–10 mg) were placed in alumina crucibles and flowed through nitrogen gas at a rate of 20 ml/min. The temperature increased from 25 to 600°C with heating rate 10°C/min. An empty alumina crucible was used as reference.

### 2.10 Rheological properties

All rheological measurements were carried out at a rotational rheometer (HAAK RS6000, Germany) coupled to a thermostatized circulating water bath (DC5‐HAAkE K15), regulated by a Peltier (HAAk UTM) Controller. Flow behaviors of 0.5% or 1% (W/V) GFPF solution were analyzed by using an internal cylinder Z34 (DIN53019) and an external cylinder Z34 (DIN53018). The rheological behaviors of 2% or 2.5% (W/V) GFPF solution were determined by with parallel plate geometry (PP20 Ti, 20 mm diameter, 1 mm gap).

#### 2.10.1 Steady shear flow behavior

The GFPF was dissolved in distilled water by magnetic stirring to obtain different concentrations samples (0.5%, 1%, and 2% w/v). The samples were stored (25°C, 12 hr) for adequately hydration in distilled water before experiments. The effects of various concentrations at 25°C and temperatures (5, 25, and 45°C) at 2% w/v were on the rheological behavior of GFPF.

The power law model was employed to explain the flow behavior of pectin.

(3) $$\tau =K{\left(\gamma \right)}^{n}$$

where *τ* represents the shear stress, γ represents the shear rate, k represents a consistency index, and n is the index of power law model.

#### 2.10.2 Linear viscoelastic region and dynamic oscillatory measurements

Strain sweeps were performed (0.01%‐1000% strain, 1 HZ) to determine the extension of the linear viscoelastic region (LVR). The LVR of GFPF was conducted for latter dynamic oscillatory measurements. The frequency sweep (0.1HZ ‐ 10HZ) was performed in strain control mode, and the selected strain was within the LVR range of all samples.

### 2.11 Antioxidant activity

The antioxidant activity of GFPF was evaluated by using DPPH and ABTS assays as described by (Shao et al., 2014) with some modification. GFPF was dissolved in distilled water to prepare different concentrations (0.1, 0.2, 0.4, 0.6, and 0.8 mg/ml) solution. Ascorbic acid was used as positive control. For DPPH assay, an aliquot of each sample (2.0 ml) was added to 2.0 ml DPPH solution (0.08 mg/ml). After incubated in the dark for 60 min, the absorbance was measured at 517 nm by UV‐5500 ultraviolet–visible spectrometer (Shimadzu, Japan). For ABTS assay, the reaction was initiated by adding 2.0 ml of ABTS solution to 2.0 ml of samples. Followed by the vortex mixing, the absorbance value of the reaction mixture was measured at 734 nm.

The ability to scavenge the DPPH and ABTS radicals was calculated according to the following equation:

(4) $$\text{Radical}\;\text{scavenging}\;\text{activity}\;\left(\%\right)=\left(1-\frac{\text{Ai}}{\text{A0}}\right)\times 100\%$$

where A_0_ is the absorbance value of DPPH or ABTS radical cations and A_i_ is the absorbance value of the sample. The IC_50_ value was defined as the concentration of GFPF required for reducing 50% of the DPPH or ABTS radicals.

### 2.12 Statistical analysis

All the data were analyzed by Excel software, and the results were expressed as the mean and standard deviation. All the figures were plotted using Origin 8.5 software.

## 3 RESULTS AND DISCUSSION

### 3.1 Chemical composition and monosaccharide analysis of GFPF

The extraction yield and chemical composition of the GFPF are summarized in Table 1. The polysaccharide extraction yield was 18.04 ± 1.81% (W/W), which was relative higher than that of *Althaea officinalis* L*.* flower (9.0%) (Tab arsa, Anvari, Joyner, Behnam, & Tab arsa, 2017) or *Lonicera japonica* flower (4.6%) (Lin et al., 2016) and was comparable with the yield of pectin extraction from apple pomace (19.8%) (Wikiera, Mika, Starzynska‐Janiszewska, & Stodolak, 2016). GFPF mainly consisted of neutral sugars (46.83 ± 3.14) and small amount of proteins (1.63 ± 0.34%). The phenol composition was also presented in GFPF with 9.49 ± 0.08 mg GAE/g. The uronic acid sugars of GFPF were 35.21 ± 0.17%, which indicated that it is a strongly acidic polysaccharide. The DE of GFPF was 32.76 ± 1.58%.

**Table 1:** Yield and chemical composition of gardenia flower polysaccharide fraction^a^

Sample	
Yield (%)	18.04 ± 1.81
Neutral sugars (%)	46.83 ± 3.14
Uronic acid (%)	35.21 ± 0.17
Protein (%)	1.63 ± 0.34
Total phenol (mgGAE/g)	9.49 ± 0.08
Degree of esterification (%)	32.76 ± 1.58%
Mw × 10^3^ (g/mol)	141.50 ± 52.09
Mn × 10^3^ (g/mol)	1.52 ± 0.52
Polydispersity (Mw/Mn)	92.20 ± 2.82
Monosaccharide composition (%)	
Galacturonic acid	41.05 ± 0.59
Galactose	17.21 ± 0.12
Glucose	16.11 ± 0.38
Arabinose	13.04 ± 0.27
Rhamnose	6.41 ± 0.10
Xylose	2.75 ± 0.20
Glucuronic acid	1.74 ± 0.02
Mannose	1.69 ± 0.05

^a^ The values given represent the average of three independent measurements.

The molecular weight distribution is an important parameter related to the rheological properties of polysaccharide. As shown in Table 1, Mw and Mn of GFPF were 141.50 ± 52.09 × 10^3^ g/mol and 1.52 ± 0.52 × 10^3^ g/mol, respectively. Polydispersity index (PDI), defined as Mw/Mn, was 92.20 ± 2.82. PDI is often used to quantify the distribution of molecular weight, and larger PDI represents a more dispersed molecular weight distribution of GFPF.

**Figure 1:**
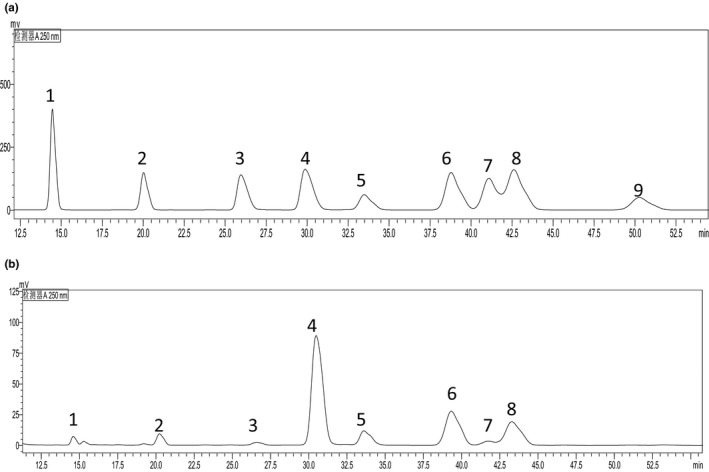
Chromatograms of PMP derivatives of monosaccharide standard samples (a) and hydrolysate of gardenia flower polysaccharide fraction (b). 1: Mannose; 2: Rhamnose; 3: Glucuronic acid; 4: Galacturonic acid; 5: Glucose; 6: Galactose; 7: Xylose; 8: Arabinose; 9: Fucose

Monosaccharide composition of GFPF was analyzed by a precolumn PMP‐derivatization HPLC method. As shown in Table 1 and Figure 1, galacturonic acid (41.05 ± 0.59%) was the major monosaccharide of GFPF. Galactose, glucose, arabinose, rhamnose, xylose, mannose, and glucuronic acid were also detected in GFPF. The result indicated that GFPF is a galacturonic acid‐rich acidic polysaccharide.

### 3.2 FT‐IR analysis

**Figure 2:**
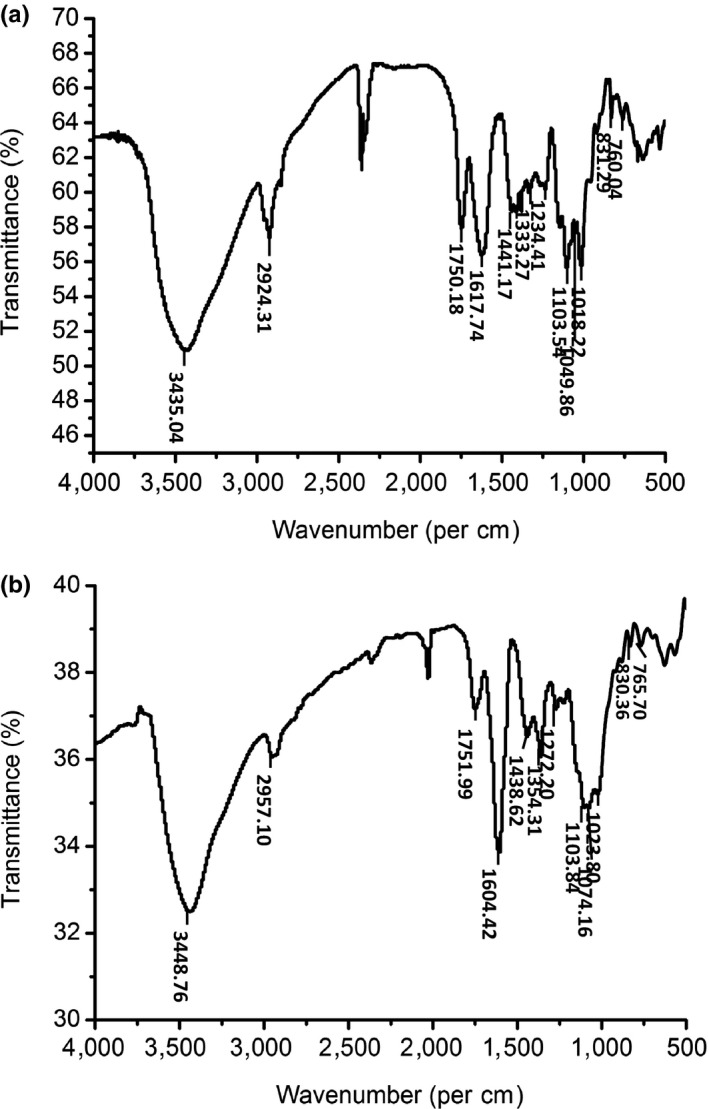
Fourier transform infrared spectra of gardenia flower polysaccharide fraction (a) and commercial pectin from apple (b)

FT‐IR spectroscopy of GFPF and pectin from apple was compared by using wavelength range from 500 cm^−1^ to 4,000 cm^−1^. As shown in Figure 2a, the strong and broad peak of GFPF was found at 3,435.04 cm^−1^, which was attributed to O‐H stretching vibration (Yang, Nisar, Hou, et al., 2018). The peak at 2,924.31 cm^‐1^ was referred to C‐H stretching from CH_2_ groups. The peak at 1,750.18 cm^‐1^ was contributed to C=O stretching vibration of ‐COOH, while the peak at 1617.74 cm^‐1^ was the C=O asymmetric stretching vibration of the free carboxylic acid or carboxylate (Manrique & Lajolo, 2002). The band area of the carboxylate stretching band (1617.74 cm^−1^) was larger than ester carbonyl groups (1,750.18 cm^−1^). Its confirms the previous DE result that GFPF was low methoxylated polysaccharide. The peaks of 1,234.41 cm^−1^, 1,333.27 cm^−1^, and 1,441.17 cm^−1^ may be due to coupling of the deformation vibrations of groups containing hydrogen atoms, including HCH, CCH, HCO, and COH (Tab arsa et al., 2017). The peaks at 1,018.22 cm^‐1^, 1,049.86 cm^−1^, and 1,103.54 cm^−1^ suggested the presence of C‐O‐C and C‐O‐H stretching vibration, which was of pyranose ring (Han et al., 2016). The peaks at 1,103.54 cm^−1^ and 1,018.22 cm^−1^ indicated that the GFPF contains uronic acid. The FT‐IR spectra of GFPF displayed a similar general pattern and exhibited similarities of the absorption patterns to that of pectin from apple (Figure 2b), suggesting that GFPF may be a pectin‐rich polysaccharide.

### 3.3 Zeta potential analysis

**Figure 3:**
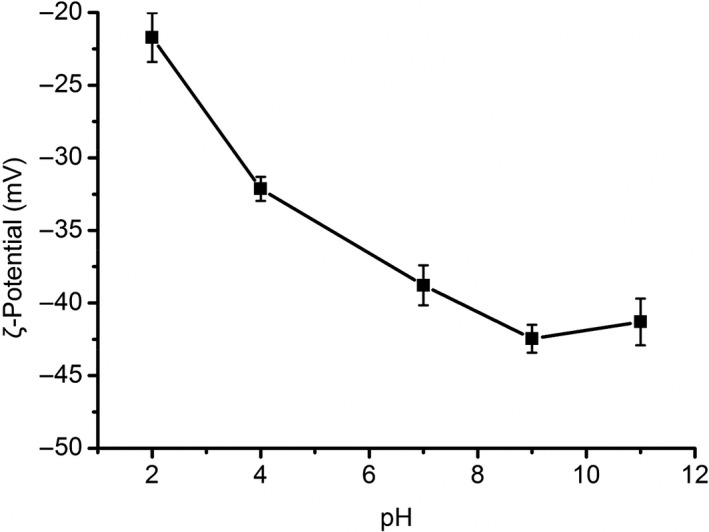
Zeta potential value of gardenia flower polysaccharide fraction (0.1 mg/ml) at various pH

As shown in Figure 3, the zeta potential value of GFPF decreased from −21.17 mV to −42.46 mV along with pH elevation from 2.0 to 11.0, which implied the presence of high fraction of hydroxyl and carboxyl radicals. This may be due to the carboxylate anion concentration increase with pH increase (Yang, Nisar, Liang, et al., 2018). However, the zeta potential values of GFPF solution were −42.46 mV and −41.30 mV at pH 9.0 and 11.0, respectively. The results indicated that the GFPF had better stability under alkaline conditions.

### 3.4 Thermal analysis

**Figure 4:**
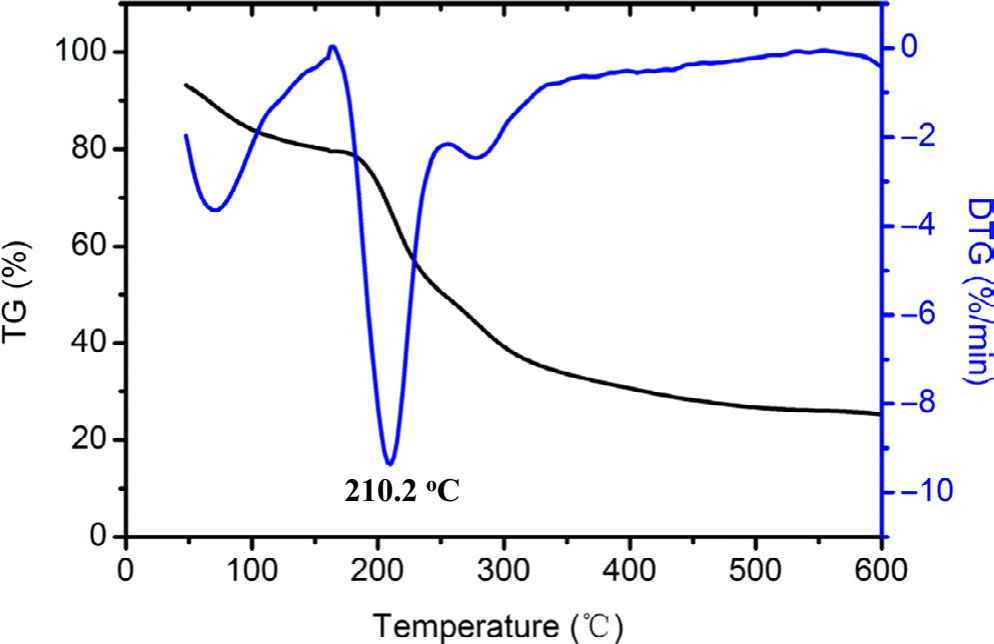
TG/DTG curve of gardenia flower polysaccharide fraction

As shown in Figure 4, the thermogravimetry (TG) and derivative thermogravimetry (DTG) curve of GFPF showed three stages: 50–190°C, 190–400°C, and 400–600°C, which was similar to the results of other pectins. The second stage, from 190 to 400°C, corresponded to ~50% weight loss, and the polysaccharide had a main weight loss peak centered at 210.2°C, which mainly attributed to the polysaccharide pyrolytic decomposition (Einhorn‐Stoll & Kunzek, 2009). The results showed that the thermal characteristics of GFPF are similar to citrus pectin (Einhorn‐Stoll & Kunzek, 2009).

### 3.5 Rheological properties

#### 3.5.1 Effect of GFPF concentration on steady shear flow behavior

**Figure 5:**
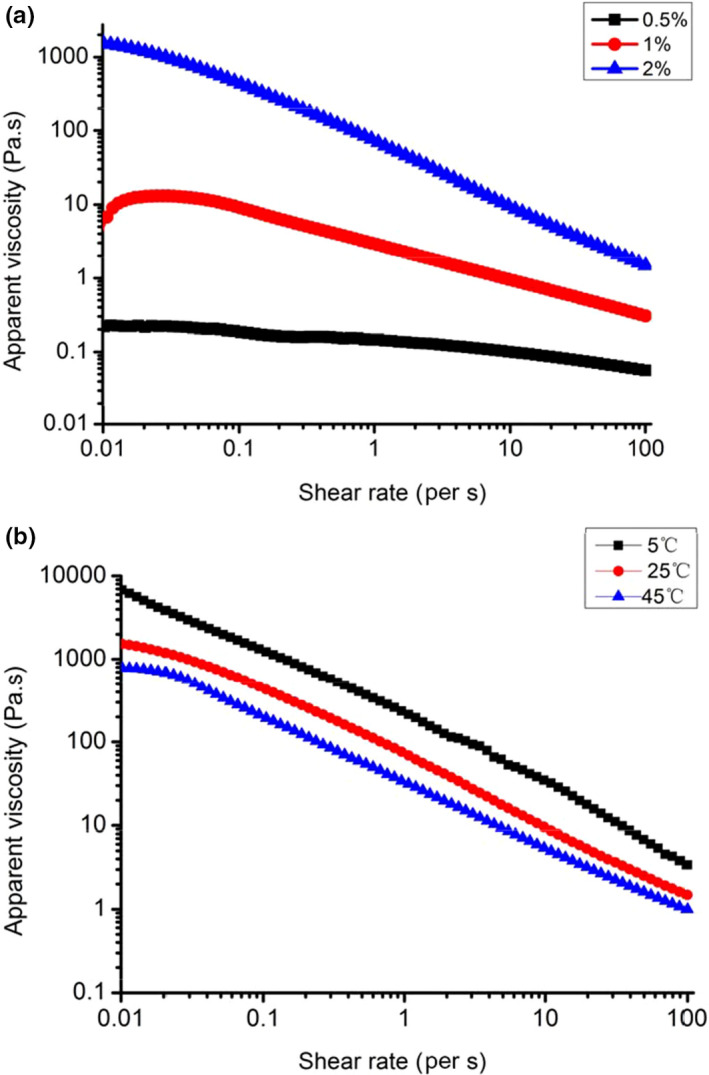
Steady shear flow curves of gardenia flower polysaccharide fraction at different concentrations under 25°C (a) and steady shear flow curves of 2% gardenia flower polysaccharide fraction at different temperatures (b)

The apparent viscosity of different concentrations (0.5%, 1%, and 2%) of GFPF solution was investigated at 25°C with the shear rate increase from 0.01 s^−1^ to100 s^−1^. As shown in Figure 5a, the apparent viscosity of GFPF increased with the concentration increase and decreased with the shear rate raising. The apparent viscosity at 2% was more than 1,000 Pa.s at the shear rate of 0.01 s^−1^ which was about 20,000 times higher than that of 0.5%. The results were well matched with other polysaccharides, such as gum from *Althaea officinalis* L*.* flower (Tab arsa et al., 2017), pectin from *Premna microphylla* Turcz leaves (Lu et al., 2019), or blueberry wine pomace (Feng, Zhou, Ashaolu, Ye, & Zhao, 2019). The increase in the viscosity has been attributed to enhance a zipping‐up process by the higher number of hydrogen bonds formed with the availability of hydroxyl groups increasing, which leads to entangle and aggregate the macromolecule chains and limits their movement (Kjøniksen, Hiorth, & Nyström, 2005).

The effect of different temperatures (5, 25, and 45°C) on the rheological properties was investigated using 2% GFPF. As expected, the apparent viscosity decreased with the temperature increase and with the shear rate raising (Figure 5b). The weakening of the electrostatic effect and the increasing of the thermodynamic effect were companied with temperature increase (Wu et al., 2018), which might led to the decrease of viscosity.

The steady rheological characteristics of GFPF solutions were described by fitting the shear rate and shear stress to power law equation. As illustrated in Table 2, the coefficients of determination (*R* ^2^) for all measured samples were above 0.93, implying that the power law model can be applied to describe the flow properties of the GFPF solutions. The consistency coefficient K increased from 0.133 to 62.683 Pa s^n^ as GFPF concentration increases from 0.5% to 2%. The K value of GFPF was similar to a pectin from the roots of *Arctium lappa* L. at 2% (k = 60.657 Pa s^n^) (Li et al., 2019) and the high methyl‐esterified pectic fraction (STW‐A) from tamarillo at 3% mixing with 50% sucrose (k = 43.979 Pa s^n^) (Nascimento, Simas‐Tosin, Iacomini, Gorin, & Cordeiro, 2016) and is more higher than that of many other pectins (Feng et al., 2019).

**Table 2:** Power law parameters of storage modulus (G′) and loss modulus(G″) for gardenia flower polysaccharide fraction solution

Condition	K	*n*	*R* ^2^
0.5%+25°C	0.133	0.856	0.999
1%+25°C	2.629	0.575	0.958
2%+25°C	62.683	0.205	0.931
2%+5°C	195.736	0.193	0.934
2%+45°C	32.624	0.232	0.982

The flow behavior index n of all tested samples was less than 1.0 (0.19–0.86) which demonstrated the pseudoplastic (shear‐thinning) nature of GFPF. The *n* value decreased from 0.856 to 0.205 with the concentration increasing from 0.5% to 2%, while the effect of temperature on flow behavior index (n) was negligible, implying that GFPF is stable below 45°C. Hydrocolloid with low *n* value has high viscosity and provides a pleasant mouthfeel (Marcotte, Taherian Hoshahili, & Ramaswamy, 2001).The results indicated that GFPF may be suitable for the application in food industry.

#### 3.5.2 Dynamic viscoelastic behavior

##### Strain sweep

**Figure 6:**
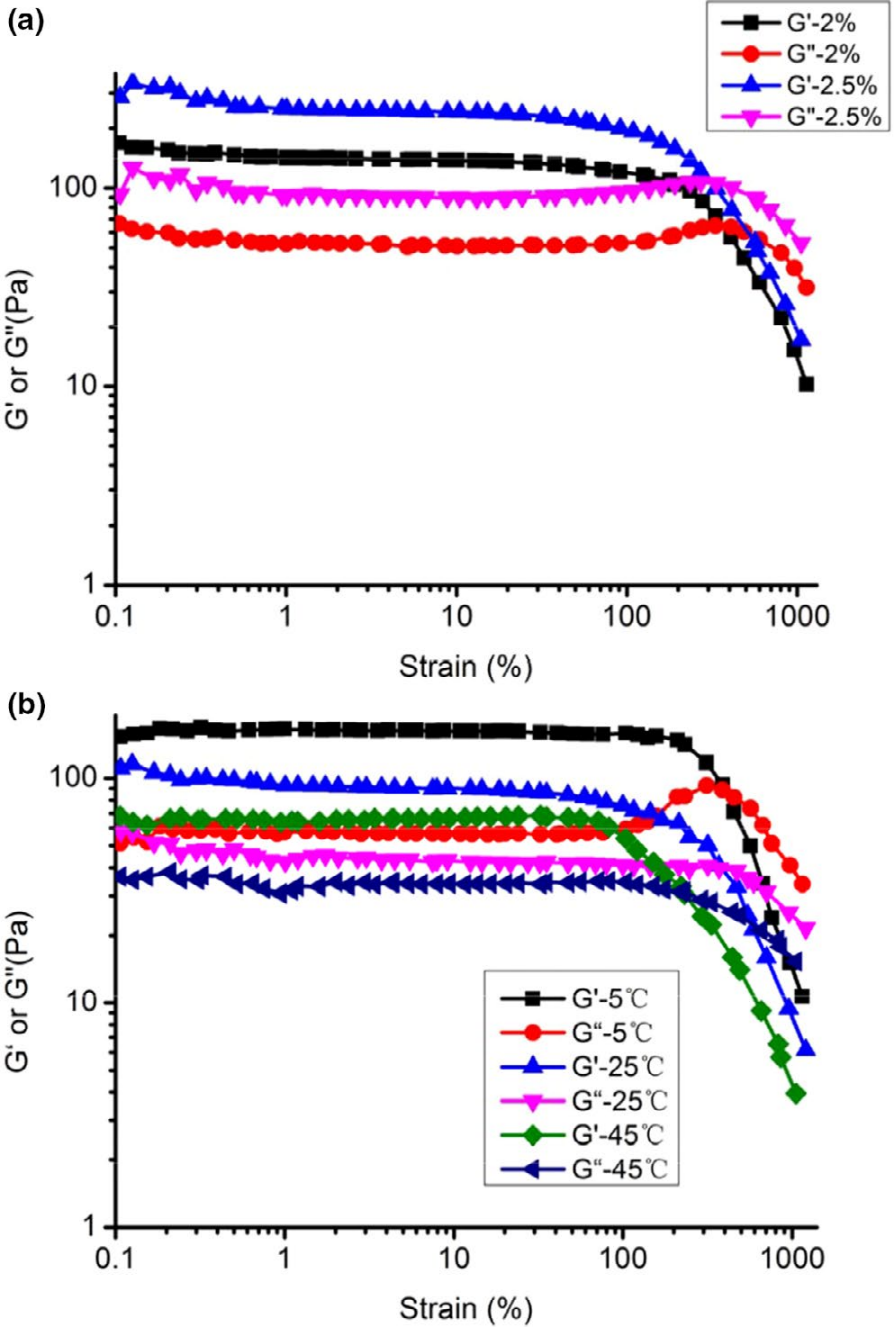
Strain sweep dependency of storage modulus (G′) and loss modulus(G″) for gardenia flower polysaccharide fraction solution at (a) the concentration of 2.0% and 2.5% under 25°C and (b) different temperatures (2% W/V)

The strain sweep tests of GFPF were carried out under different concentrations (2% and 2.5%) or different temperatures (5, 25, and 45°C), and the results are shown in Figure 6. The storage modulus (G′) and loss modulus (G′′) values of GFPF 2.5% were higher than that of 2%, indicating higher rigidity of the system at higher concentrations (Figure 6a). Conversely, both moduli values decreased with temperature increase from 5 to 45°C (Figure 6b). According strain sweep tests results, strain 1% was chosen as experimental shape variable for frequency sweep tests.

##### Frequency sweep

**Figure 7:**
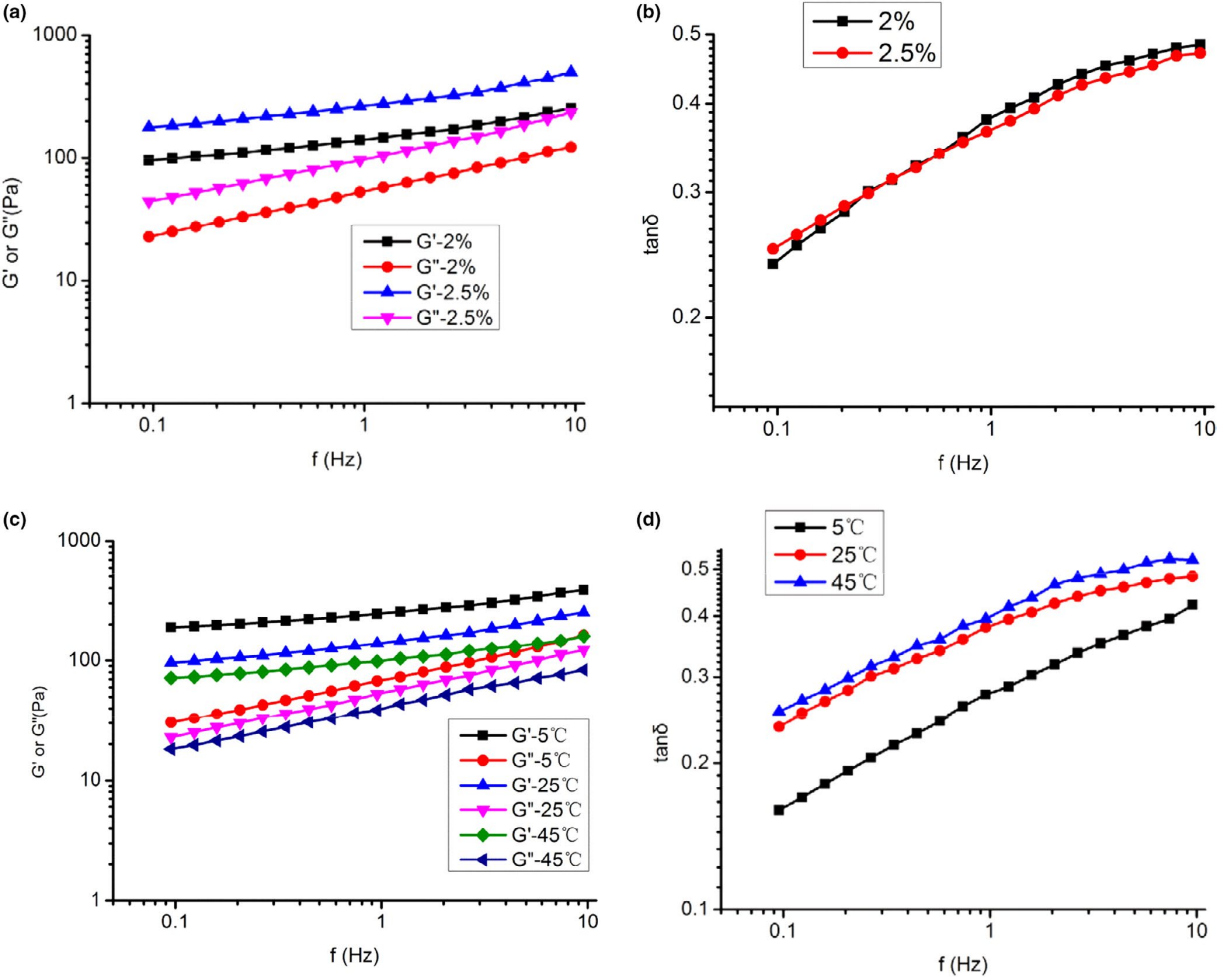
Frequency sweep of gardenia flower polysaccharide fraction solution. (a) Viscoelastic moduli (G′ and G″) at concentration of 2.0% and 2.5% under 25°C; (b) damping factor (tan δ, G″/G′) at concentration of 2.0% and 2.5% under 25°C; (c) viscoelastic moduli (G′ and G″) at different temperatures (2% W/V); (d) damping factor (tan δ, G″/G′) at different temperatures (2% W/V)

The frequency sweep test was conducted to investigate the mechanical response of GFPF gel over the frequency of 0.1 HZ‐10 HZ. The results of G′, G′′, and damping factor (tan δ, G″/G′) are presented in Figure 7.

The value of G' and G" of 2.5% was much higher than those of 2%, moduli almost 5 times higher than that of 2% at 0.1 HZ, indicating that the gel strength was concentration dependent (Figure 7a). Besides, the value of G' and G" tends to converge at higher frequency and tan δ increased from 0.23 to 0.5 with frequency argument (Figure 7b). The results indicated that the formation of a network between interchain and intrachain (Yang, Nisar, Liang, et al., 2018) and gel properties became weaker in higher frequency.

The effect of temperature on its viscoelasticity is shown in Figure 7c. The values of *G′* and *G″* decreased with the temperature increase*.* The tan δ increased with frequency argument, and the value of *5*°C was lower than that of 25°C or 45°C (Figure 7d). The results demonstrated that the gel became weak with the temperature increase.

### 3.6 Antioxidant activity of GFPF

**Figure 8:**
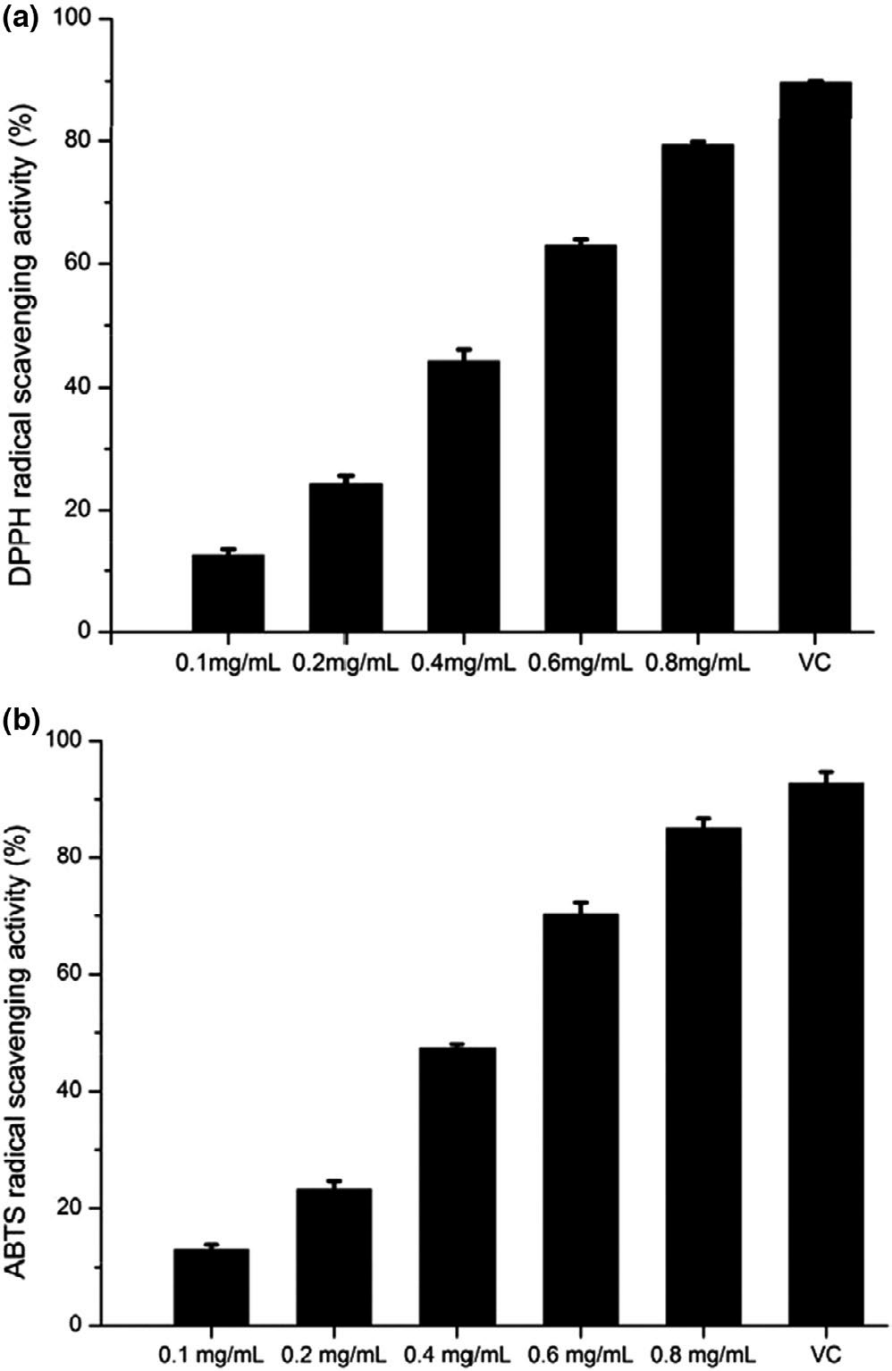
Antioxidant activities of gardenia flower polysaccharide fraction. (a) DPPH radical scavenging activity; (b) ABTS radical scavenging activity; ascorbic acid (16 μg/ml) was used as positive control

To evaluate the antioxidant activity of GFPF, its DPPH or ABTS free radical scavenging activity is investigated. As illustrated in Figure 8, GFPF demonstrated the free radical scavenging capability in a concentration‐dependent manner at the concentration range of 0.1–0.8 mg/ml. The DPPH free radical scavenging activity increased from 12.51% to 79.32% along with concentration from 0.1 to 0.8 mg/ml (Figure 8a). The DPPH free radical scavenging IC_50_ for GFPF was 0.47 mg/ml. As for ABTS free radical, GFPF exhibited the similar dose‐dependent scavenging effect as for DPPH (shown in Figure 8b). The IC_50_ for ABTS free radical scavenging was 0.43 mg/ml. However, its DPPH or ABTS free radical scavenging capability was lower than that of ascorbic acid (16 μg/ml).

Compared with many other pectins, such as pectin extracted from apple pomace and citrus peel by subcritical water (IC_50_ > 4.6 mg/ml) (Wang, Chen, & Lu, 2014) and fig (*Ficus carica* L.) skin (IC_50_ > 5.0 mg/ml) (Gharibzahedi, Smith, & Guo, 2019; 222), GFPF had a relatively higher antioxidant activity with lower IC_50_ (<0.47 mg/ml). It was reported that the interaction between pectin and phenolic compounds displayed a synergistic effect on antioxidant activity (Mercado‐Mercado, de la Rosa, & Alvarez‐Parrilla, 2020). Total phenol content of GFPF was 9.49 mg GAE/g which may be the reason for its high antioxidant activity.

## 4 CONCLUSIONS

This study demonstrated that gardenia flower is a new resource of low methoxy pectin (DE = 32.76 ± 1.52%) and the extraction yield was 18.04 ± 1.81%%. It was rich in galacturonic acid (41.05 ± 0.59%). The flow and viscoelastic properties of GFPF were dependent on concentration and temperature. The apparent viscosity was fit with power law model (*R* ^2^ > 0.93) and showed a good pseudoplastic behavior. The dynamic viscoelastic behaviors of GFPF displayed gel‐like behaviors with the G*′* that was higher than G*′′*. Furthermore, GFPF showed high antioxidant activity with low IC_50_ for DPPH and ABTS free radical scavenging. The results suggest that GFPF can be a very promising source of high‐quality low methoxyl pectin and for food and nonfood applications.

## CONFLICT OF INTEREST

The authors declare no conflict of interest.

## References

[fsn31612-bib-0001] Blumenkrantz, N. , & Asboe‐Hansen, G. (1973). New method for quantitative determination of uronic acids. Analytical Biochemistry, 54(2), 484–489. 10.1016/0003-2697(73)90377-1 4269305

[fsn31612-bib-0002] Bradford, M. M. (1976). A rapid and sensitive method for the quantitation of microgram quantities of protein utilizing the principle of protein‐dye binding. Analytical Biochemistry, 72(1), 248–254. 10.1016/0003-2697(76)90527-3 942051

[fsn31612-bib-0003] Buchholt, H. C. , Christensen, T. M. I. E. , Fallesen, B. , Ralet, M.‐C. , & Thibault, J.‐F. (2004). Preparation and properties of enzymatically and chemically modified sugar beet pectins. Carbohydrate Polymers, 58(2), 149–161. 10.1016/j.carbpol.2004.06.043

[fsn31612-bib-0004] Chan, S. Y. , Choo, W. S. , Young, D. J. , & Loh, X. J. (2017). Pectin as a rheology modifier: Origin, structure, commercial production and rheology. Carbohydrate Polymers 161, 118–139. 10.1016/j.carbpol.2016.12.033 28189220

[fsn31612-bib-0005] Cho, E.‐H. , Jung, H.‐T. , Lee, B.‐H. , Kim, H.‐S. , Rhee, J.‐K. , & Yoo, S.‐H. (2019). Green process development for apple‐peel pectin production by organic acid extraction. Carbohydrate Polymers, 204, 97–103. 10.1016/j.carbpol.2018.09.086 30366548

[fsn31612-bib-0006] Dai, J. , Wu, Y. , Chen, S.‐W. , Zhu, S. , Yin, H.‐P. , Wang, M. , & Tang, J. (2010). Sugar compositional determination of polysaccharides from *Dunaliella salina* by modified RP‐HPLC method of precolumn derivatization with 1‐phenyl‐3‐methyl‐5‐pyrazolone. Carbohydrate Polymers, 82(3), 629–635. 10.1016/j.carbpol.2010.05.029

[fsn31612-bib-0007] Dranca, F. , & Oroian, M. (2018). Extraction, purification and characterization of pectin from alternative sources with potential technological applications. Food Research International, 113, 327–350. 10.1016/j.foodres.2018.06.065 30195527

[fsn31612-bib-0008] Einhorn‐Stoll, U. , & Kunzek, H. (2009). Thermoanalytical characterisation of processing‐dependent structural changes and state transitions of citrus pectin. Food Hydrocolloids, 23(1), 40–52. 10.1016/j.foodhyd.2007.11.009

[fsn31612-bib-0009] Feng, L. , Zhou, Y. , Ashaolu, T. J. , Ye, F. , & Zhao, G. (2019). Physicochemical and rheological characterization of pectin‐rich fraction from blueberry (*Vaccinium ashei*) wine pomace. International Journal of Biological Macromolecules, 128, 629–637. 10.1016/j.ijbiomac.2019.01.166 30708018

[fsn31612-bib-0010] Gharibzahedi, S. M. T. , Smith, B. , & Guo, Y. (2019). Ultrasound‐microwave assisted extraction of pectin from fig (Ficus carica L.) skin: Optimization, characterization and bioactivity. Carbohydrate Polymers, 222, 114992 10.1016/j.carbpol.2019.114992 31320048

[fsn31612-bib-0011] Giacomazza, D. , Bulone, D. , San Biagio, P. L. , & Lapasin, R. (2016). The complex mechanism of HM pectin self‐assembly: A rheological investigation. Carbohydrate Polymers, 146, 181–186. 10.1016/j.carbpol.2016.03.046 27112864

[fsn31612-bib-0012] Han, Q. H. , Wu, Z. L. , Huang, B. , Sun, L. Q. , Ding, C. B. , Yuan, S. , … Yuan, M. (2016). Extraction, antioxidant and antibacterial activities of *Broussonetia papyrifera* fruits polysaccharides. International Journal of Biological Macromolecules, 92, 116–124. 10.1016/j.ijbiomac.2016.06.087 27370746

[fsn31612-bib-0013] Han, W. Y. , Meng, Y. H. , Hu, C. Y. , Dong, G. R. , Qu, Y. L. , Deng, H. , & Guo, Y. (2017). Mathematical model of Ca2+ concentration, pH, pectin concentration and soluble solids (sucrose) on the gelation of low methoxyl pectin. Food Hydrocolloids, 66, 37–48. 10.1016/j.foodhyd.2016.12.011

[fsn31612-bib-0014] Hosseini, S. S. , Khodaiyan, F. , & Yarmand, M. S. (2016). Optimization of microwave assisted extraction of pectin from sour orange peel and its physicochemical properties. Carbohydrate Polymers, 140, 59–65. 10.1016/j.carbpol.2015.12.051 26876828

[fsn31612-bib-0015] Kjøniksen, A.‐L. , Hiorth, M. , & Nyström, B. (2005). Association under shear flow in aqueous solutions of pectin. European Polymer Journal, 41(4), 761–770. 10.1016/j.eurpolymj.2004.11.006

[fsn31612-bib-0016] Lapornik, B. , Prošek, M. , & Golc, W. A. (2005). Comparison of extracts prepared from plant by‐products using different solvents and extraction time. Journal of Food Engineering, 71(2), 214–222. 10.1016/j.jfoodeng.2004.10.036

[fsn31612-bib-0017] Li, K. D. , Zhu, L. L. , Li, H. , Zhu, Y. Q. , Pan, C. , Gao, X. D. , & Liu, W. (2019). Structural characterization and rheological properties of a pectin with anti‐constipation activity from the roots of *Arctium lappa* L. Carbohydrate Polymers, 215, 119–129. 10.1016/j.carbpol.2019.03.051 30981336

[fsn31612-bib-0018] Lin, L. Y. , Wang, P. P. , Du, Z. Y. , Wang, W. C. , Cong, Q. F. , Zheng, C. P. , … Shao, C. (2016). Structural elucidation of a pectin from flowers of *Lonicera japonica* and its antipancreatic cancer activity. International Journal of Biological Macromolecules, 88, 130–137. 10.1016/j.ijbiomac.2016.03.025 27000440

[fsn31612-bib-0019] Lu, J. K. , Li, J. J. , Jin, R. C. , Li, S. F. , Yi, J. J. , & Huang, J. Y. (2019). Extraction and characterization of pectin from *Premna microphylla* Turcz leaves. International Journal of Biological Macromolecules, 131, 323–328. 10.1016/j.ijbiomac.2019.03.056 30857960

[fsn31612-bib-0020] Manrique, G. D. , & Lajolo, F. M. (2002). FT‐IR spectroscopy as a tool for measuring degree of methyl esterification in pectins isolated from ripening papaya fruit. Postharvest Biology and Technology, 25(1), 99–107. 10.1016/S0925-5214(01)00160-0

[fsn31612-bib-0021] Marcotte, M. , Taherian Hoshahili, A. R. , & Ramaswamy, H. (2001). Rheological properties of selected hydrocolloids as a function of concentration and temperature. Food Research International, 34(8), 695–703. 10.1016/S0963-9969(01)00091-6

[fsn31612-bib-0022] Marić, M. , Grassino, A. N. , Zhu, Z. , Barba, F. J. , Brnčić, M. , & Rimac, B. S. (2018). An overview of the traditional and innovative approaches for pectin extraction from plant food wastes and by‐products: Ultrasound‐, microwaves‐, and enzyme‐assisted extraction. Trends in Food Science & Technology, 76, 28–37. 10.1016/j.tifs.2018.03.022

[fsn31612-bib-0023] Mercado‐Mercado, G. , de la Rosa, L. A. , & Alvarez‐Parrilla, E. (2020). Effect of pectin on the interactions among phenolic compounds determined by antioxidant capacity. Journal of Molecular Structure, 1199, 126967 10.1016/j.molstruc.2019.126967

[fsn31612-bib-0024] Nascimento, G. E. , Simas‐Tosin, F. F. , Iacomini, M. , Gorin, P. A. J. , & Cordeiro, L. M. C. (2016). Rheological behavior of high methoxyl pectin from the pulp of tamarillo fruit (*Solanum betaceum*). Carbohydrate Polymers, 139, 125–130. 10.1016/j.carbpol.2015.11.067 26794955

[fsn31612-bib-0025] Rover, M. R. , Johnston, P. A. , Lamsal, B. P. , & Brown, R. C. (2013). Total water‐soluble sugars quantification in bio‐oil using the phenol–sulfuric acid assay. Journal of Analytical and Applied Pyrolysis, 104, 194–201 10.1016/j.jaap.2013.08.004

[fsn31612-bib-0026] Shan, M. Q. , Yu, S. , Yan, H. , Guo, S. , Xiao, W. , Wang, Z. Z. et al (2017). A Review on the phytochemistry, pharmacology, pharmacokinetics and toxicology of geniposide, a natural product. Molecules, 22(10), 1689 10.3390/Molecules22101689 PMC6151614 28994736

[fsn31612-bib-0027] Shao, Q. S. , Deng, Y. M. , Liu, H. B. , Zhang, A. L. , Huang, Y. Q. , Xu, G. Z. et al (2014). Essential oils extraction from *Anoectochilus roxburghii* using supercritical carbon dioxide and their antioxidant activity. Industrial Crops and Products, 60, 104–112. 10.1016/j.indcrop.2014.06.009

[fsn31612-bib-0028] Tabarsa, M. , Anvari, M. , Joyner, H. S. , Behnam, S. , & Tabarsa, A. (2017). Rheological behavior and antioxidant activity of a highly acidic gum from *Althaea officinalis* flower. Food Hydrocolloids, 69, 432–439. 10.1016/j.foodhyd.2017.02.009

[fsn31612-bib-0029] Trujillo‐Ramírez, D. , Lobato‐Calleros, C. , Román‐Guerrero, A. , Hernández‐Rodríguez, L. , Alvarez‐Ramirez, J. , & Vernon‐Carter, E. J. (2018). Complexation with whey protein hydrolysate improves cacao pods husk pectin surface active and emulsifying properties. Reactive and Functional Polymers, 123, 61–69. 10.1016/j.reactfunctpolym.2017.12.011

[fsn31612-bib-0030] Wan, L. , Chen, Q. , Huang, M. , Liu, F. X. , & Pan, S. Y. (2019). Physiochemical, rheological and emulsifying properties of low methoxyl pectin prepared by high hydrostatic pressure‐assisted enzymatic, conventional enzymatic, and alkaline de‐esterification: A comparison study. Food Hydrocolloids, 93, 146–155. 10.1016/j.foodhyd.2019.02.022

[fsn31612-bib-0031] Wang, F. , Miao, M. B. , Xia, H. , Yang, L. G. , Wang, S. K. , & Sun, G. J. (2017). Antioxidant activities of aqueous extracts from 12 Chinese edible flowers in vitro and in vivo. Food Nutrition Research, 61(1), 1265324 10.1080/16546628.2017.1265324 28326000 PMC5328308

[fsn31612-bib-0032] Wang, X. , Chen, Q. R. , & Lu, X. (2014). Pectin extracted from apple pomace and citrus peel by subcritical water. Food Hydrocolloids, 38, 129–137. 10.1016/j.foodhyd.2013.12.003

[fsn31612-bib-0033] Wikiera, A. , Mika, M. , Starzynska‐Janiszewska, A. , & Stodolak, B. (2016). Endo‐xylanase and endo‐cellulase‐assisted extraction of pectin from apple pomace. Carbohydrate Polymers, 142, 199–205. 10.1016/j.carbpol.2016.01.063 26917391

[fsn31612-bib-0034] Wu, Y. , Guo, R. , Cao, N. N. , Sun, X. J. , Sui, Z. Q. , & Guo, Q. B. (2018). A systematical rheological study of polysaccharide from *Sophora alopecuroides* L. seeds. Carbohydrate Polymers, 180, 63–71. 10.1016/j.carbpol.2017.10.007 29103522

[fsn31612-bib-0035] Xiao, W. , Li, S. , Wang, S. , & Ho, C.‐T. (2017). Chemistry and bioactivity of *Gardenia jasminoides* . Journal of Food and Drug Analysis, 25(1), 43–61. 10.1016/j.jfda.2016.11.005 28911543 PMC9333430

[fsn31612-bib-0036] Yang, X. , Nisar, T. , Hou, Y. , Gou, X. , Sun, L. , & Guo, Y. (2018). Pomegranate peel pectin can be used as an effective emulsifier. Food Hydrocolloids, 85, 30–38. 10.1016/j.foodhyd.2018.06.042

[fsn31612-bib-0037] Yang, X. , Nisar, T. , Liang, D. , Hou, Y. , Sun, L. , & Guo, Y. (2018). Low methoxyl pectin gelation under alkaline conditions and its rheological properties: Using NaOH as a pH regulator. Food Hydrocolloids, 79, 560–571. 10.1016/j.foodhyd.2017.12.006

[fsn31612-bib-0038] Yin, F. , & Liu, J.‐H. (2018). Research and application progress of *Gardenia jasminoides* . Chinese Herbal Medicines, 10(4), 362–370. 10.1016/j.chmed.2018.09.001

[fsn31612-bib-0039] Yoo, S. H. , Fishman, M. L. , Savary, B. J. , & Hotchkiss, A. T. Jr (2003). Monovalent salt‐induced gelation of enzymatically deesterified pectin. Journal of Agricultural and Food Chemistry, 51(25), 7410–7417. 10.1021/jf030152o 14640592

[fsn31612-bib-0040] Yuliarti, O. , Chong, S. Y. , & Goh, K. K. T. (2017). Physicochemical properties of pectin from green jelly leaf (*Cyclea barbata* Miers). International Journal of Biological Macromolecules, 103, 1146–1154. 10.1016/j.ijbiomac.2017.05.147 28577980

[fsn31612-bib-0041] Zhang, H. , Feng, N. , Xu, Y. T. , Li, T. X. , Gao, X. M. , Zhu, Y. , … Wu, H. H. (2017). Chemical constituents from the flowers of wild *Gardenia jasminoides* J. ELLIS. Chemistry Biodiversity, 14(5), e1600437 10.1002/cbdv.201600437 28130824

[fsn31612-bib-0042] Zhang, L.‐S. , Wang, Y.‐L. , Liu, Q. , Zhou, C.‐X. , Mo, J.‐X. , Lin, L.‐G. , & Gan, L.‐S. (2018). Three new 3,4‐seco‐cycloartane triterpenoids from the flower of *Gardenia jasminoides* . Phytochemistry Lettler, 23, 172–175. 10.1016/j.phytol.2017.11.006

